# Assessment of emergency medicine residents: a systematic review

**Published:** 2017-02-24

**Authors:** Isabelle N. Colmers-Gray, Kieran Walsh, Teresa M. Chan

**Affiliations:** 1 Faculty of Medicine and Dentistry, University of Alberta, Alberta, Canada; 2 BMJ Learning, BMJ, London, UK; 3 McMaster University, Ontario, Canada

## Abstract

**Background:**

Competency-based medical education is becoming the new standard for residency programs, including Emergency Medicine (EM). To inform programmatic restructuring, guide resources and identify gaps in publication, we reviewed the published literature on types and frequency of resident assessment.

**Methods:**

We searched MEDLINE, EMBASE, PsycInfo and ERIC from Jan 2005 – June 2014. MeSH terms included “assessment,” “residency,” and “emergency medicine.” We included studies on EM residents reporting either of two primary outcomes: 1) assessment type and 2) assessment frequency per resident. Two reviewers screened abstracts, reviewed full text studies, and abstracted data. Reporting of assessment-related costs was a secondary outcome.

**Results:**

The search returned 879 articles; 137 articles were full-text reviewed; 73 met inclusion criteria. Half of the studies (54.8%) were pilot projects and one-quarter (26.0%) described fully implemented assessment tools/programs. Assessment tools (n=111) comprised 12 categories, most commonly: simulation-based assessments (28.8%), written exams (28.8%), and direct observation (26.0%). Median assessment frequency (n=39 studies) was twice per month/rotation (range: daily to once in residency). No studies thoroughly reported costs.

**Conclusion:**

EM resident assessment commonly uses simulation or direct observation, done once-per-rotation. Implemented assessment systems and assessment-associated costs are poorly reported. Moving forward, routine publication will facilitate transitioning to competency-based medical education.

## Introduction

### Background

In the past three decades there has been a movement within medical education toward the practice of Competency Based Medical Education (CBME).^1^–^4^ This movement harkens back to mid-20^th^ century where educational systems were being changed to ensure pre-specified discrete learner outcomes.^5^ Since the 1980s, a revival of this movement has given rise to various bodies and initiatives within medical education, namely: The General Medical Council (GMC) guidance of the United Kingdom;^1^ the Accreditation Council for Graduate Medical Education (ACGME) Competencies and Milestones project in the United States;^2^ and the Educating Future Physicians of Ontario (EFPO) and CanMEDS competency initiatives in Canada.^3^,^4^

Traditionally, the predominant model of postgraduate training has emphasized experience and time spent in the clinical setting^6^ with additional final assessments (i.e., written exams, oral exams, and Objective Structured Clinical Exams [OSCEs]).^7^ The current shift in educational systems towards emphasizing learner-oriented outcomes, such as competencies in various skills, has created a need for more robust (validated and reliable) tools and systems to assess learners.^8^ An assessment tool is a single structured scale, form, rubric or exam used to measure performance, knowledge, skills or abilities; whereas assessment programs and systems involve a formalized and multi-faceted approach used to evaluate and offer feedback to learners. Further, there is increasing interest in measuring clinical performance in the workplace, and ensuring that a learner is able to achieve the “Does” level at the peak of Miller’s Pyramid (which outlines a learner’s progression from “Knows” at the base of the pyramid, through “Knows How” and “Shows,” to reach “Does”).^9^

### Importance

Within emergency medicine (EM) training, learners must develop a wide range of skills and competencies outlined by CanMEDS and ACGME.^10^,^11^ Since the introduction of CanMEDS 2005,^4^ available assessment tools relevant to EM in the Western world have been described in recent consensus reports and summaries.^12^–^14^ Still, the actual prevalence of the use of these tools has not been reported in the literature.

The growing emphasis on competency assessment in medical training increases the need for resources required for assessment.^14^,^15^ Assessment tools vary in cost: contrast, for example, the resources required to create, administer, and mark a pen-and-paper MCQ exam, with the costs of training, personnel, simulation mannequins, equipment, and software programs required for a simulation-based assessment.^16^ The cost and true value of a tool is determined in the context of outcomes (using, for example, cost-effectiveness, cost-benefit or cost-feasibility analyses).^17^–^19^ In medical education, however, cost is infrequently measured. Determining overall impact and value of an assessment strategy adopted for competency assessment demands measuring not only outcomes but also associated resources or costs.

Measuring clinical competence of EM residents will require educators to understand the breadth of existing assessment tools and systems in order to identify next steps in transitioning to CBME (including implementation of existing tools or systems and development of new ones). Literature on costs associated with assessment tools/systems or effectiveness analyses will be useful in guiding planning for (re)allocation of resources to implement competency based education. To date, there has been no detailed description of how frequently different types of assessment systems are being used in Western training programs, nor has there been a review of cost reporting associated with assessment tools or systems.

### Goals of this investigation

To quantify what assessment systems are in use and to summarize the regularity of their use, we systematically searched the published literature to determine 1) the type and availability of published assessment tools or systems and 2) their frequency of use in emergency medicine resident assessment. As a secondary outcome, we summarized information on the cost of these assessments.

## Methods

### Study design

This study is a systematic review of published literature. It does not require research ethics board approval. Our study was conducted according to an a priori protocol agreed upon by all authors and reporting follows PRISMA guidelines.^20^

### Methods and measurements

The literature search was developed in collaboration with a research librarian and included EMBASE, Medline, ERIC, and PsychInfo; these were most likely to retrieve our articles of interest, as well as abstracts from relevant EM and medical education conferences. We searched for MeSH terms such as “resident,” “assessment,” and “evaluation,” then used published filters to limit our search to EM.^21^–^23^

The search was limited to studies in the English language, published January 2005 through June 2014 (i.e., in the period following the release of the CanMEDS 2004 competencies). A sample search strategy is included in Appendix A.

Two authors (ICG, TMC) independently reviewed titles and abstracts for suitability, and then further reviewed full-text studies for inclusion. Inclusion criteria required full text studies or abstracts of Emergency Medicine trainees (residents) in North America, Europe, Australia or New Zealand, and a report of at least one of the primary outcomes of interest. We excluded studies in undergraduate medical students or fellows only, non-EM residency programs, and studies published before 2005. As our objective was to review assessment programs and tools that were actually used (rather than list the available types, which has been done elsewhere),^12^,^14^,^24^–^27^ we excluded review/summary articles and consensus reports. Data abstraction followed our pre-specified protocol and included demographics of the study population, teaching centre, assessment tools, scope of the program, frequency of assessment, and associated costs/resources.

Definitions for validity have changed greatly over the past century.^28^ More recent definitions of validity center on the interpretations or actions that result from a tool, as well as the appropriateness of the tool for a particular context, and have moved away from viewing validity as an inherent property of a test. In addition, the appropriateness of a test encompasses notions of construct validity (i.e., measuring what a test purports to measure), structural validity (i.e., correlation with other similar constructs), and content validity (i.e., relevance of construct to the test’s intended use),^28^ which are captured by the unified criteria for construct validity outlined by Messick.^29^

We applied the Messick criteria to gauge to what extent the reported assessment tools had undergone testing to demonstrate evidence of construct validity. Messick outlines a framework of six levels (or aspects) for which the overall construct validity of a tool can be gauged: content (relevance, representativeness, and technical quality of the items/tasks assessing the domains of interest); substantive (theoretical rationale and observed evidence for consistencies in responses); structural (how well the scoring structure reflects the domain being assessed); generalizability (how well score properties and interpretations can be extended to other populations, settings or tasks); external (how well scores correlate with other external measures of other tests); and consequential aspects (intentional or unintentional social impact of the score as basis for action or change). Although the Messick criteria are not structured on a hierarchy of validity, the more criteria a tool demonstrates, the stronger the argument for global construct validity of that tool, and the more meaningful it becomes. In an effort to characterize the strength of validity evidence for the tools found in our review, we defined a tool as demonstrating “good” construct validity if it had been tested on at least two different aspects of Messick’s validity framework. Since the goal of this study was to quantify the prevalence of various assessment tools and programs reported in the literature, we did not evaluate each publication for its quality as a study in and of itself, as it would have had little or no bearing on our study results and their interpretation.

### Outcomes

The two primary outcomes of this study are: 1) the types of assessment tools used, including assessment programs; and 2) the number of assessments per resident in whichever timeframe reported by a study. A secondary outcome is the presence of any report of cost for a described assessment system.

### Analysis

Findings were tabulated and summarized using descriptive statistics calculated in Microsoft Excel (2011). Where possible, median and interquartile range (IQR) were presented. Multicentre trials were counted as individual centres when calculating program duration and number of participants. Frequency of assessment calculations assumed one-month rotations; “one-off” or pilot studies were not included in overall frequency of assessment calculations. We used a post-hoc sensitivity analysis to test the impact of our assumption of one-month rotations by assuming a three-month rotation (i.e., when extrapolating the annual assessment frequency for a tool reported per rotation, we multiplied the number of assessments by four, rather than 12, to test our assumption). Given the descriptive nature of our study design, we did not conduct comparative analyses.

### Cost reporting

Estimation and analysis of costs in the medical education literature is notoriously challenging.^30^ A reasonable approach to identifying and measuring costs suggested in the literature is the ingredients method.^18^–^31^ Dividing ingredients up into a number of different categories may facilitate identifying key components of cost. Most attention should be paid to ingredients that make up most of the costs (such as equipment, resources, and personnel, including faculty or staff physicians). A list of ingredients relevant to determining costs related to assessment tools are outlined in Box 1.^18^,^19^,^31^

Box 1 **Key cost ingredients and reporting methods to estimate costs related to medical education assessment systems**

**Cost Ingredients:**

- Personnel (e.g., faculty, staff)
- Facilities
- Equipment and consumables
- Learner inputs
- Tool development and validation
- Software, programs and IT support
- Patient/actor time and participation
- Maintenance costs
- Other(s)

**Cost reporting:**

- Cost ingredients
- Overall cost
- Cost per learner
- Highlight upfront or investment costs

For the purposes of this study, our cost analysis involved abstracting reports of resources (i.e., costs) required for an assessment tool/system; however, since our goal was to identify the presence (and not quantification) of resource/cost reporting, we did not conduct further analyses.

## Results

**Figure 1:**
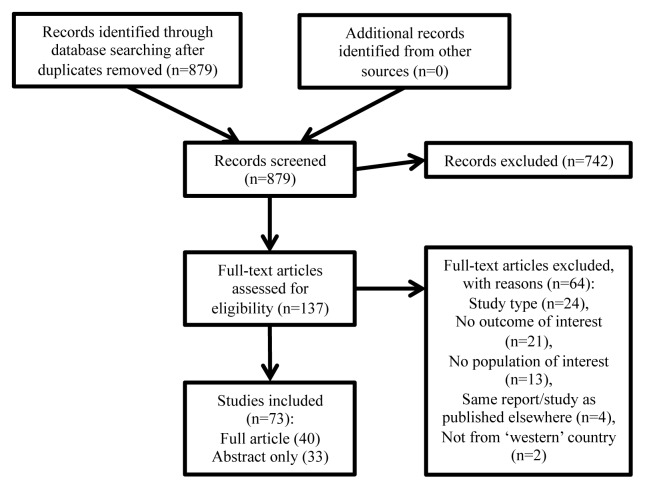
Study selection

The literature search returned 879 articles after removal of duplicates (Figure 1). We excluded 742 articles based on screening of title and abstract. Of the remaining 137 articles that went to full-text review, 64 were subsequently excluded, most commonly for lacking an outcome of interest (n=21), the study type (i.e., papers which were summary or consensus reports; n=17) or lacking our population of interest (n=13). Other reasons are detailed in Figure 1. In total, 73 reports met our inclusion criteria: 40 full-text articles^32^–^71^ and 33 abstracts.^72^–^104^

### Study demographics

Over 80% of reports originated from the United States and a limited portion (14%) were from Canada (Table 1). There were three multicentre studies, conducted in two,^33^ four^105^ and eight^71^ different American sites. The median duration of residency program was three years, with five-year programs representing 20% of studies. Some studies also included non-EM residents: Pediatric EM, Internal Medicine, and/or Family Medicine. Fifty-four studies reported the number of residents in a program or participants in a study, which were 37 (IQR: 30–49) and 30 (IQR: 15–52), respectively.

We used the Messick framework of construct validity (involving six criteria described in the Methods section) to evaluate the strength of validity evidence of the reported assessment tools. The median number of Messick’s validity criteria reported per tool was one (IQR: 1–2). Thirty-five reports (47.9%) fulfilled two or more of Messick’s criteria, suggesting roughly half of assessment tools had attempted to demonstrate multiple forms of validity evidence (Table 1). Detailed information on the demographics of each included study is available online (eSuppl 1).

### Assessment tools

Studies described a variety of assessment tools that differed in scope (Table 1). Over half of reports (n=40) were pilot projects of assessment tools (including tool development, validation, and testing). Only 19 studies reported a fully implemented assessment program. Other studies were designed to evaluate aspects of how the assessment tool performed in comparison to other methods or its correlation with other factors.

Seventy-two studies reported a total of 111 assessment tools, with a median of one (IQR: 1–2) tool reported per study. These tools comprised 12 main categories, plus “other” (Appendix B). The most commonly reported tools used to assess EM residents were written or standardized tests (n=21), simulation-based assessments (n=21), and direct observation with an assessment checklist (n=19; including Standardized Direct Observation Tool (SDOT) [n=4], Mini-Clinical Evaluation Exercise (Mini-CEX) [n=2] and others, including novel tools [n=13]). We found no report of chart-simulated recall as a method of assessment. The least frequently reported assessment tools (with only two reports of each) were: patient surveys, In-Training Evaluation Reports (ITERs)/end-of-rotation assessments, procedure logs, and reflective portfolios.

A total of 19 studies reported fully implemented assessment tools and/or programs (see Appendix B). Six studies described fully implemented assessment programs and further explored the validity and/or reliability of their methods in the following ways: the correlation between self-, peer-, and faculty-assessments when leading a simulation;^82^ how well the tool (ITER) correlates with CanMEDS competencies;^50^ resident assessments from nursing colleagues;^106^ the degree of correlation between direct observation evaluations and quarterly evaluations;^62^ the correlation between faculty ratings and objective structured clinical exam (OSCE) scores;^102^ and correlation between OSCE scores and subsequent ACGME scores.^68^ Five studies reported assessment programs used for the following: implementing curricula in pediatric EM;^40^ high-fidelity simulation;^80^ pain management;^58^ international EM rotations,^56^ and communication.^66^ Two studies described new methods of assessing competency of incoming residents.^47^,^103^ Two reports describe assessment programs for a senior resident teaching role/rotation.^48^,^101^ Other reports described the implementation of the SDOT program;^42^ a theme-based hybrid simulation model;^60^ an end-of-rotation examination for the pediatric intensive care unit;^34^ and the use of exams from a national EM question bank for resident assessment.^36^

Interestingly, one study of Pediatric EM fellows reported an absence of assessment on their knowledge of medical care costs.^54^

In total, 25 studies reported how a program evolved over time: three programs were simplified or scaled back in some way after the initial pilot^44^,^48^,^59^ and 11 programs were expanded, scaled up, or exported to other programs following the initial pilot.^32^,^42^,^48^,^51^,^55^,^56^,^78^,^81^,^84^,^101^,^105^ One program was scaled back in some aspects and expanded in others.^48^

### Frequency of assessment

There were 39 studies reporting information on how often residents received any form of assessment (eSuppl 2). The frequency of assessment ranged from daily to once during residency. The most common frequencies reported were twice per month/rotation (n=6), once annually (n=4) and three times ever (n=3). Daily (n=2), bi-weekly (n=3) and weekly (n=3) feedback within a month/rotation were also reported.

**Figure 2:**
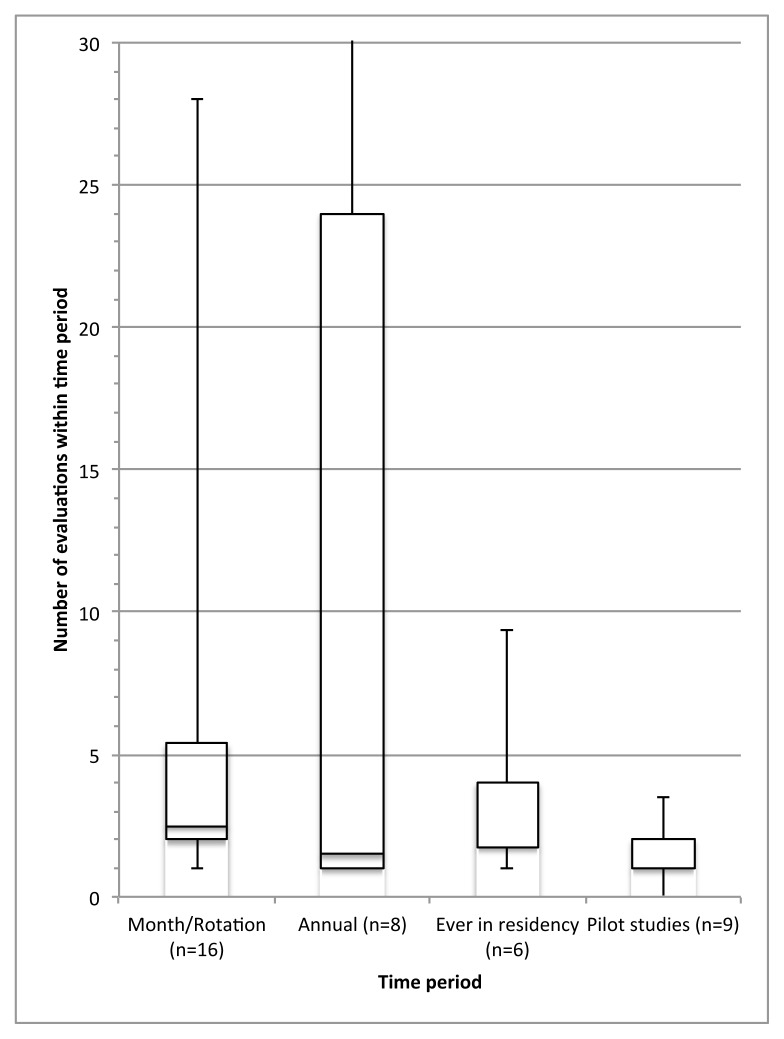
Median number of assessment of residents by time interval reported for each tool

The reported assessment frequency per resident per tool is summarized in Figure 2. The median number of assessments was stratified by the time period over which the assessment tool was used: within the entire residency program (median: 4 [IQR: 1.75–4], n=6); per annum (1.5 [1–24], n=8); and per month/rotation (2.5 [2–5.4], n=16). Assuming the assessment frequency reported continued throughout residency, the overall median number of assessments per resident annually was twice monthly (median: 24 [IQR: 1.1–48], n=30). In pilot studies of assessment tools, the median number of assessments was one (IQR: 1–2, n=9).

As a sensitivity analysis to test our assumption of one-month rotations, we calculated a separate frequency for studies reporting assessments “per rotation” (n=13), using a three-month assumption for duration of rotation. With this assumption, the median annual assessment frequency was 12 (IQR: 8–32) among the studies reporting “per rotation” assessments. Using this same three-month rotation assumption, the overall median number of assessments was 20 (IQR 8–48.5), a change of 16% from the previous model assuming one-month rotations (median 24 assessments per annum).

The most frequently used assessment tools were: daily encounter cards;^93^,^99^ direct observation;^48^,^78^ oral case presentations;^86^ and 360 degree/multisource feedback.^48^ Of studies reporting higher assessment frequency, only one^48^ was a fully implemented program.

Lower frequency of assessment was associated with being a pilot or “one-off” study. Tools used for more infrequent assessments (four or less per year) include: written exams;^53^,^67^,^76^,^98^ direct observation (e.g. SDOT, DOTs, mini-CEX);^42^,^47^,^62^,^68^ simulation;^37^,^47^,^98^ OSAT;^68^,^103^ OSCE;^68^,^91^ and global/faculty assessment.^47^,^107^

### Cost reporting

We reported the presence of cost reporting for a given assessment tool or system within our 73 studies. Though no article presented the exact cost of their assessment tool or curriculum, two provided estimates.^39^,^70^

Brazil et al. report that adding four MiniCEX assessments for a 20-intern (PGY1) ED rotation extrapolates to costing $80,000 (AUS) annually.^39^ To assess communication and interpersonal skills of 12 residents that involved unannounced standardized patients, Zabar et al. report compensating eight actors $25/h for training and 17 ED interviews, with an approximated total of $2,037 to $3,100 USD.^70^

Some studies noted the use of various resources, however it was difficult to determine which of these resources already existed (i.e., no additional cost) or were required specifically for the tool.

## Discussion

Our systematic search found 73 studies, which we used to determine the type and frequency of tools or systems used to assess emergency medicine residents. The most commonly reported assessment tools were written or standardized tests, simulation-based assessments, and direct observation. Assessment frequency was reported by half of the studies and ranged from daily to once ever in residency. The median frequency was twice per one-month rotation. No study provided the total cost of a given assessment tool, though two provided estimates.

It is not surprising that, despite an extensive literature search, we found fewer than 20 studies that describe a fully implemented assessment tool or program. Though the concept of outcomes-based medical education has grown since the 1980s, a concrete set of competencies for EM residents was only introduced via CanMEDS in 2005 (Canada) and the EM Milestone Project in 2012 (USA).^4^,^108^

It is possible there is still a lag in published reports of assessment tools and systems used by EM residency programs. The high number of published abstracts found by our literature search could be an indicator of full article publications in the coming months and years. It is also possible, however, that residency programs lack support, incentive, impetus, or precedent to publish their assessment systems. If so, this must be addressed and encouraged, especially now, while program directors and other educators implement novel assessment systems in the transition to CBME (such as the Canadian CanMEDS Competency By Design frameworks).

Over half of the reports in our review describe pilot (i.e., “one-off”) studies. Clearly, there is an abundance of literature describing, testing, and validating novel assessment tools; what is missing, however, is follow-up from such studies on higher levels of outcome, including the learner-level (e.g., achievement on standardized exams, advancement or promotion within a residency program or graduating sooner), patient-level (e.g., improved satisfaction with care, time waiting to be seen), or system-level (e.g., readmission rates, productivity, medical errors, near-misses, etc).^109^ As we continue to adopt CBME and its educational approach, innovation will be key to building capacity in sound competency assessment.

Studies in this review largely omitted cost reporting. Estimates of costs related to assessment tools were provided by only two studies. Determining cost(s) associated with an assessment tool is paramount to its existence; without securing resources (including funding), an assessment system will be difficult to sustain. Medical education researchers should be strongly encouraged to determine the value of an intervention – beyond an instrument’s correlation with other learning tools, whether learners and/or faculty enjoy it, and so on. The move towards CBME is already in progress and, by determining costs, administrators and directors can anticipate how they must (re)allocate resources to support this approach to learner assessment.

Cost analyses of medical education programs are notoriously difficult; competency assessment systems are no exception.^30^ There are insufficient precedent, experience and, to a certain degree, interest among medical educators in conducting cost analyses of proposed assessment tools. A few recent publications can help guide non-economists in conducting cost or resource analyses.^17^–^19^,^30^ In the context of resource analyses for learner assessment tools, the “ingredients” method, which compiles a list of resources required, is useful to tabulate total cost. Common categories that have emerged in the literature and are relevant to learner assessment tools or systems are summarized in Box 1. Further, we suggest cost be reported in three ways: 1) ingredients; 2) total cost; and 3) per-learner cost. Should there be a large upfront investment cost required (for example, purchasing of new equipment for simulation training), reporting the “initial investment cost” will provide context for interpreting the three aforementioned costs.

### Limitations

We may not have captured the full breadth of information available on this topic, for two main reasons. First, as with all systematic reviews, it is possible our search did not capture the full extent of indexed literature. However, we did capture a large number of abstracts, which suggests a broad search. As well, given our interest in English language studies published after 2004, the vast majority of published studies would likely be indexed and captured. Secondly, and most importantly, peer-review publication of assessment methods and systems is not done systematically across all residency programs. This limitation was anticipated *a priori* and our study intentionally highlights the paucity of publications of assessment tools and systems. Capturing unpublished information on resident assessment, such as through a survey of program directors or review of program websites, was a delimitation of our study and out of the scope of our systematic review but would be valuable to pursue with future studies. Another limitation is our inability to assess costs. We did abstract cost metrics, however these are challenging to approximate or report. For example, “ingredients” such as hours spent by faculty members, running a computer system, or hospital supplies are difficult to quantify but are key in implementing and establishing a CBME system. Lastly, we made a reasonable assumption that a rotation was one-month, which allowed us to calculate an overall median frequency of assessment. If the average duration of rotations is longer than one-month then our assumption is an overestimation of assessment frequency. Despite our bias toward the “best case scenario” of rotations of one month, assessment still occurred rather infrequently. Our sensitivity analysis, which checked the one-month assumption by assuming three-month rotations, showed minimal change in the overall frequency of annual assessment (24 vs 20 assessments annually).

### Lessons learned

As medical educators develop and validate methods of learner assessment, their research should be held to the same standards as any other area of rigorous scientific inquiry; this necessitates (peer-reviewed) publication and distribution of knowledge and experiences as well as related costs. Through this, we can develop assessment methods that are feasible, resource-effective and, hence, sustainable. CBME presents a great opportunity to galvanize our nation’s community of medical educators. We hope that by pointing out the deficits in the present literature we can encourage our community to share their innovations and contribute to the community as a whole. Key take-home points for medical educators are summarized in Box 2.

Box 2 **Key findings and next steps for resident assessment**

**Key findings:**

- Assessment programs and tools are poorly reported and rarely published
- Multiple tools exist to assess different competencies
- Most residents receive assessments twice a month; the frequency of unofficial and formative feedback is unclear
- Pilot programs lack data on system-level outcomes

**Recommendations for next steps:**

- Publication of assessment tools, systems and programs is essential
- Adapting and improving existing tools and systems can streamline (rather than duplicate) efforts and resources
- Cost reporting is a key element in determining the impact of an assessment program or tool

Substantial work in the area of residency assessment exists, but few programs have reported successful implementation of a rigorous assessment system in EM. Moreover, even fewer programs have reported costs of such residency assessment systems. As we move forward in the era of CBME, there will be great need for reports of assessment tools and systems, including frequency of assessment, costs, and higher-level outcomes.

## Supplementary Information





## Figures and Tables

**Table 1:** Characteristics of included papers

Characteristic	Studies reporting (N=73)	Median (IQR)
**Country of origin**	73	

USA	59 (81%)	
Canada	10 (14%)	
Europe	2 (3%)	
Australia	2(3%)	
New Zealand	0 (0%)	

**Scope of study**	73	

Pilot project	40 (55%)	
Correlation/validation	9 (12%)	
Comparative	57%)	
Assessment programs	19 (26%)	

Duration of residency*	65	3 (IQR: 3–4)

3 years	44 (59%)	
4 years	15 (20%)	
5 years	15 (20%)	

# residents**	55	

in program	24 (41%)	37 (IQR: 30–49)
in study	31 (56%)	30 (IQR: 15–52)

# validity criteria demonstrated***	73	1 (IQR: 1–2)

0	8 (11%)	
1	30 (41%)	
2	23 (32%)	
≥3	12 (16%)	

* 65 publications comprising 74 programs

** 55 publications comprising 59 programs

*** Number of construct validity levels demonstrated for each assessment tool (based on 6 criteria of the Messick Framework for global construct validity)

## Appendix A EMBASE search strategy

1. exp Medical Students/ or exp Medical Education/ or exp Physicians/ or exp Specialization/ or emergency medicine.mp. or clinical experience/ or learning experience/ or * “clinical teaching (health professions)”/ or exp graduate medical education/ or medical education/ or student experience/ or “residen”.ti,ab. (632160)
2. exp Evaluation/ or exp Nongraded Student Evaluation/ or exp Summative Evaluation/ or exp Student Evaluation/ or
  evaluation.mp. or exp Formative Evaluation/ or *course objectives/ or *course organization/ or curriculum evaluation/ or
  *instructional effectiveness/ or *instructional material evaluation/ or *program evaluation/ or *program validation/
  (1294683)
3. exp Evaluation/ or “assess*”.mp. (2880369)
4. 2 or 3 (3815273)
5. 1 and 4 (161227)
6. limit 5 to (english language and yr=“2005 -Current”) (119495)
7. emergency.af,ti,ab. (356722)
8. 6 and 7 (15404)
9. 6 not 8 (104091)
10. exp academic achievement/ or assess*.ti. or evaluat*.ti. (807699)
11. exp residency education/ or medical education/ (193122)
12. medical resident*.mp. or exp resident/ or (resident or residents).ti,ab. (120878)
13. 11 and 12 (19566)
14. 10 and 13 (2229)
15. limit 14 to human (1670)
16. limit 15 to (english language and yr=“2005 -Current”) (1036)
17. needs assessment.mp. or exp needs assessment/ or exp dentistry/ or dentistry.mp. (147386)
18. 16 not 17 (972)
19. Emergency ward/ or emergency medicine/ or emergency nurse practitioner/ or emergency nursing/ or emergency
  patient/ or emergency physician/ or emergency health service/ or exp emergency/ or ((emergenc* or trauma) adj1 (hospital* or service* or room* or ward or wards or unit or units or department* or physician* or doctor* or nurs* or
  accident*)).mp. or triage.mp. (227223)
20. 18 and 19 (95)
21. 18 not 20 (877)

## Appendix B Description of fully implemented assessment programs and tools

**Table:**

Study (author, year)	Location	# Residents or participants	Program duration (ys)	Type of tool	Brief study description	Total #tools	Tool type(s) used	Assessment Frequency (time period:#)	Cost reporting present?	Messick criteria demonstrated**
Akhtar, 2010	USA	Participants (EM): 42	3	Impact Assessment	Post-PICU rotation exam in EM & Pediatrics residents	1	Written/standardized exam	Rotation: 1	No	2
Beeson, 2006	USA	“Variable”	-	Tool description	Development of national EM question bank & exam in US	1	Written/standardized exam	Ever: “Multiple times”	No	3
Burnette, 2009	USA	PGY1 (37), PGY2 (42), PGY3 (16)	3	Curriculum description	Implementation & impact of online PEM curriculum on pre/post curriculum test scores	1	Written/standardized exam	-	No	1, 2, 3, 5
Clark, 2010*	Canada (Vancouver)	-	5	Curriculum description	Evaluation of high fidelity simulation program	1	Simulation	-	No	3, 4, 6
Cooper, 2012*	USA (Indiana)	Participants: 76	3	Correlation study	Correlation between self, peer and faculty assessments of leading simulation cases	1	360-degree/multisource feedback	Monthly: 2	No	1, 2
Dorfsma n, 2009	USA (Pittsburgh)	Participants: PGY1 (3), PGY2 (28), PGY3 (1)	3	Curriculum description	Implementation of SDOT program for EM residents	1	Direct observation (novel tool: adapted CORD-EM SDOT tool)	Ever: 1 (in PGY2)	No	0
Hauff, 2014	USA (Michigan)	Total incoming PGY1: 28	4	Tool description	Competency assessment of incoming interns in EM	3	Direct observation (novel tool: milestonebased clinical skills assessment tool), Simulation, Other (EM milestones global evaluation form)	Ever: 4 (in PGY1)	No	2, 3, 4
Ilgen, 2011	USA (Boston)	Total PGY4: 15	4	Curriculum description	Experience with ‘resident-asteacher’ curriculum (teaching senior role)	2	Direct observation (novel tool: based on residenttailored learning objectives using a ‘teach the teacher’ model), 360-degree/multisource feedback	Rotation: weekly	No	2, 5
Kassam, 2014	Canada (Calgary)	-	5	tool development/validation	Retrospective description of items and validation of linking to CanMEDS	1	ITER/end of rotation assessment	Rotation: 1	No	1, 2, 6
McIntosh, 2012	USA (Jacksonville, FL)	-	3	Curriculum description	Development and assessment of international EM curriculum	2	Oral/verbal exam, Reflective portfolio	Rotation: 2	No	1, 2
Motov, 2011	USA (Brooklyn, NY)	-	3	Curriculum description	Pain management curriculum	4	Written/standardized exam, OSAT, Others (pre-and posttests; customized SDOT-PAIN scale)	Rotation: 2 weekly	No	0
Noeller, 2008	USA	All residents: 38	3	Curriculum description	Testing & evaluation of a theme based hybrid simulation model	2	Written/standardized exam, Simulation	Rotation: 2	No	4, 5
Pavlic, 2014*	USA (U of Michigan, Ann Arbor)	-	4	Curriculum description	Retrospective study of nursing feedback to residents	1	360-degree/multisource feedback	-	No	1, 2
Ryan, 2010	USA (New York Hospital Queens, Flushing NY)	All residents: 30 (10 per year)	3	Curriculum description	4-year observational study of direct observation vs. quarterly evaluations	2	Direct observation (novel tool: assessment of competencies during a single patient encounter), ITER/end of rotation assessment (same tool but globally applied)	Quarterly: 21	No	1, 2, 4
Sampsel, 2014*	Canada (Ottawa)	All residents: 45	5	Curriculum description	Clinical Teaching Team program development and implementation	4	Oral/verbal exam, Direct observation (novel tool: “direct observation”), Daily encounter cards, Other (targeted clinical encounters)	Rotation: 1/3 of shifts	No	1, 2, 5
Shih, 2013*	USA	Total PGY1 residents over 5 years: 36 (avg 7 per year)	3	Correlation study	Correlation between faculty ratings and OSCE exam scores	1	OSCE	-	No	1, 2, 3
Sullivan, 2009	USA	PGY1 (10) PGY2 (8) PGY3 (8)	3	Curriculum description	Introduction and development of a communication curriculum	2	Direct observation (novel tool: communication skills checklist), Other (videotape-facilitated self assessment)	-	No	1, 5
Wagner, 2013*	USA (Michigan)	-	3	Curriculum description	Use of a standard form to assess milestones during EM1 orientation sessions	1	OSAT	Annually: 1	No	1, 6
Wallenstein, 2010	USA (Atlanta)	All PGY1: 18	3	Correlation study	Ability of early OSCE to predict ACGME core competency scores	3	OSCE, Direct observation (mini CEX), Direct observation (SDOT)	Annually: 1	No	1, 2

* = abstract only

** Messick levels of validity evidence coding: 0 = None met (and no alternative paradigm used); 1 = Structural validity; 2 = Content validity; 3 = Substantive validity; 4 = External validity; 5 = Generalizability validity; 6 = Consequential validity; 0 = none reported

Abbreviations: ACGME = American Council of Graduate Medical Education; CORD-EM = Council Of Emergency Medicine Residency Directors; EM = Emergency Medicine; ITER = In-Training Evaluation Report; Mini-CEX = Mini-Clinical Evaluation Exercise; OSAT = Objective Structured Assessment of Technical skills; OSCE = Objective Structured Clinical Exam; PGY = Post-Graduate Year (i.e., residency year); SDOT = Standardized Direct Observation Tool
