# Supplementary material for: Assessment of emergency medicine residents: a systematic review
Source: Can Med Educ J. 2017 Feb 24;8(1):e106–22. (PMC5344063)
Supplement: Supplementary file 1 [file cmej-08-106-s001.pdf]

## eSuppl 1: Study characteristics

| Study (author, year) | Location | Program duration (yrs) | Number of residents** | Messick criteria demonstrated*** |
|---|---|---|---|---|
| Abu-Laban, 2013 | Canada (British Columbia, University of British Columbia) | 5 | Participants: 12 | 2, 6 |
| Adler, 2011 | USA (Illinois, University of Chicago) | 3(n=1), 4(n=1) | Participants across 2 sites: 69 | 3, 4 |
| Aghera, 2012* | USA (New York, Brooklyn Maimonides Medical Centre) | 3 | Participants: 15 | 1,5 |
| Ahn, 2011* | USA (Illinois, University of Chicago) | ? | not specified | 6 |
| Akhtar, 2010 | USA (Florida, University of Florida) | 3 | Participants (EM): 42 | 0 |
| Ali, 2013* | USA (Georgia, Emory University) | ? | not specified | 5 |
| An-Grogan,2013* | USA (Illinois, Chicago) | ? | not specified | 5 |
| Barlas, 2011* | USA (New York, New York Hospital Queens) | 3 | All residents: 60 | 5 |
| Barsuk, 2009 | USA (Illinois, Northwestern University Feinberg School of Medicine) | 4 | EM and IM participants: 103 | 4 |
| Beeson, 2006 | USA (Multicentre) | ? | variable | 0 |
| Blouin, 2006 | Canada (Ontario, Queen's University) | 5 | All years: 30 | 0 |
| Bohrn, 2014* | USA (Illinois, WellSpan York Hospital) | 3 | 21 residents | 1 |
| Bounds, 2013 | USA (Multicentre) | 3(n=3), 5(n=1) | PGY2 (30),<br>PGY3 (39),<br>PGY4/PGY5 (3) | 1, 4 |
| Brazil, 2012 | Australia (Queensland, Royal Brisbane and Women's Hospital) | 3 | All PGY1: 20 | 5, 6 |
| Burnette, 2009 | USA (Multicentre) | 3 | PGY1 (37),<br>PGY2 (42),<br>PGY3 (16) | 2 |
| Carrière, 2009 | Canada (Quebec, Université de Montréal) | 5 | PGY1 (21) PGY2 (21) Seniors (11) | 3 |
| Chan, 2014* | Canada (Ontario, McMaster University) | 5 | PGY1 and PGY2: 14 | 1, 2, 3, 5 |
| Christian, 2012* | USA (Illinois, Cook County Hospital Stroger) | 5 | Participants only (45):<br>PGY2 (2),<br>PGY3 (30),<br>PGY4 (10),<br>PGY5 (3) | 3, 4, 6 |
| Clark, 2010* | Canada (Vancouver, University of British Columbia) | 5 | not specified | 1, 2 |
| Cloutier, 2013* | USA (Portland, Oregon Health and Science University) | 3 | not specified | 0 |
| Cooper, 2012 | USA (Indiana, Indiana University School of Medicine) | 3 | Participants: 76 | 2, 3, 4 |
| Datta, 2012* | USA (New York, New York Hospital Queens) | 3 | Participants: 29 | 2, 5 |
| Dorfsman, 2009 | USA (Pennsylvania, University of Pittsburgh) | 3 | Participants: PGY1 (3), PGY2 (28), PGY3 (1) | 1, 2, 6 |
| Flowerdew, 2012 | England (London, Imperial College) | ? | not specified | 1, 2 |

| | | | | |
|---|---|---|---|---|
| Franc, 2012 | Canada (Alberta, University of Alberta) | 5 | Participants: 25 | 0 |
| Frederick, 2011 | USA (Illinois, University of Illinois College of Medicine at Peoria) | 3 | All PGY3 (between 1994-2005): 85 (mean 7 per year) | 4, 5 |
| Gallagher, 2013* | USA (Illinois, Northwestern University Feinberg School of Medicine) | 4 | Participants: all 24 junior residents; sample of 6 senior residents | 1, 2 |
| Girzadas, 2007 | USA (Illinois, Advocate Christ Medical Center) | 3 | All residents (44):<br>incoming EM1 (11)<br>EM1 (11)<br>EM2 (11)<br>EM3 (11) | 1, 2, 4 |
| Hauff, 2014 | USA (Michigan, University of Michigan) | 4 | Total incoming PGY1: 28 | 1, 2, 5 |
| Hogan, 2012* | USA (Illinois, University of Chicago) | 4 | Participants across 6 residency programs | 1, 2, 3 |
| Howes, 2011* | USA (Chicago) | 3 | Participants: 24 | 1, 5 |
| Ilgen, 2011 | USA (Boston) | 4 | Total PGY4: 15 | 1, 6 |
| Jang, 2013 | USA (California) | 4 | Participants: 127 | 1, 2 |
| Jhun, 2014* | USA | 5 | Total residents: 67; Participants: 54 | 1, 2, 6 |
| Kassam, 2014 | Canada (Calgary) | 5 | not specified | 1, 3 |
| Kim, 2009 | Canada (Ottawa) | 5 | Participants: PGY1 (32), PGY3 (28) | 1, 3 |
| Kusmiesz, 2011* | USA (Pennsylvania) | ? | not specified | 4 |
| Kyaw, 2012 | England (London) | 3 | not specified | 1, 2, 3 |
| LaMantia, 2009 | USA (8 centres across the country) | 3 (n=6),<br>4 (n=2) | not specified | 1, 2, 3 |
| Ledrick, 2009 | USA (Ohio) | 3 | All residents (35):<br>PGY1 (12)<br>PGY2 (12)<br>PGY3 (11) | 1, 2, 3, 4, 5, 6 |
| Ledrick, 2013* | USA (Ohio) | 3 | Total between 2006 and 2012: PGY1 (81),<br>PGY2 (83),<br>PGY3 (79) | 4 |
| Lee, 2010* | Canada (Ottawa) | 5 | All PGY1: 12 | 1, 3, 5 |
| Lee, 2012 | USA | 3 | All PEM fellows: 240 | 2 |
| Leech, 2013* | USA (Florida) | 3 | Participants: 14 | 3 |
| Leone, 2011* | USA (Oregon, Chicago) | 4 | Participants: 45 | 3 |
| Lifchez, 2012 | USA (Baltimore) | 3 | Participants: 7 | 5 |
| Mamtani, 2014* | USA (Pennsylvania, Philadelphia) | 4 | not specified | 0 |
| Marinelli, 2012* | USA (Chicago) | 4 | Participants: 45 | 1 |
| McGrath, 2014* | USA (Columbus, OH) | 3 | Participants: 35 | 5 |
| McIntosh, 2012 | USA (Jacksonville, University of Florida College of Medicine, Jacksonville) | 3 | not specified | 6 |
| McLaughlin, 2007 | USA (Albuquerque, New Mexico) | 3 | Participants: 27 | 2, 4 |

| | | | | |
|---|---|---|---|---|
| Minnigan, 2012* | USA (Indianapolis, IN) | 3 | All PGY2: 12 | 1 |
| Motov, 2011 | USA (Brooklyn, NY) | 3 | not specified | 1, 2 |
| Murray, 2014* | USA (Ann Arbor, Michigan) | 4 | Participants: 52 | 3 |
| Nelson, 2013* | USA (Missouri, Children's Mercy Hospital and Clinic) | 3 | Participants: 55 | 5 |
| Noble, 2007 | USA (Multicentre) | 4 | Participants: 12 | 2 |
| Noeller, 2008 | USA (Multicentre) | 3 | All residents: 38 | 1, 2 |
| O'Connor, 2014* | USA (Multicentre) | ? | not specified | 0 |
| Pavlic, 2014* | USA (Michigan, University of Michigan) | 4 | not specified | 1 |
| Reisdorff, 2006 | USA (Michigan, Michigan State University) | 3 | Participants: 19: PGY2(9), PGY3 (10) | 1 |
| Ryan, 2010 | USA (New York, New York Hospital Queens) | 3 | All residents: 30 (10 per year) | 1, 3 |
| Sampsel, 2014* | Canada (Ontario, University of Ottawa) | 5 | All residents: 45 | 6 |
| Samuel, 2009 | Australia (multicentre) | 5 | Participants: 47 | 1 |
| Scher, 2011 | USA (Connecticut, University of Connecticut Health Center) | 5 | Participants (EM): 18 | 2 |
| Schwaab, 2011 | USA (Ohio, Ohio State University) | 3 | all residents: 36 participants: 27 | 1, 2 |
| Shih, 2013* | USA (New Jersey, Morristown Medical Center) | 3 | Total PGY1 residents over 5 years: 36 (avg 7 per year) | 5 |
| Sullivan, 2009 | USA (Missouri, University of Missouri) | 3 | PGY1 (10)<br>PGY2 (8)<br>PGY3 (8) | 3 |
| Thundiyil, 2010 | USA (Florida, University of Florida) | 3 | PGY3 Participants (2002-2006): 51 | 5 |
| Wagner, 2013* | USA (Michigan, Central Michigan University) | 3 | not specified | 2 |
| Wallenstein, 2010 | USA (Georgia, Emory University) | 3 | All PGY1: 18 | 5 |
| Williams, 2009 | USA (Texas, San Antonio Military Medical Center) | ? | not specified | 3 |
| Wittels, 2013* | USA (Massachusetts, Harvard Affiliated Programs at Brigham and Women's Hospital and Massachusetts General Hospital) | ? | not specified | 3 |
| Zabar, 2009 | USA (New York, New York University School of Medicine) | ? | All PGY2: 15 | 3, 6 |

Note:

* = abstract only

** = number of participants if number of residents unclear

*** Messick criteria: 1 = Structural validity; 2 = Content validity; 3 = Substantive validity; 4 = External validity; 5 = Generalizability validity; 6 = Consequential validity; 0 = none reported

PGY = post-graduate year (i.e., residency level)
