# Supplementary material for: Assessment of emergency medicine residents: a systematic review
Source: Can Med Educ J. 2017 Feb 24;8(1):e106–22. (PMC5344063)
Supplement: Supplementary file 2 [file cmej-08-106-s002.pdf]

eSuppl 2: Assessment tools and frequency of assessment reported among included studies

Study (author, year)	Study type	Scope of study	Assessment tool(s) reported	Frequency (per time period: # assessments)
Abu-Laban, 2013	pilot of tool / curriculum	Pilot	Written/standardized exam	Rotation: 2
Adler, 2011	tool development / validation	Pilot	Simulation-based	
Aghera, 2012*	Comparative study	Comparative study	Simulation-based	
Ahn, 2011*	Impact Assessment	Pilot	Other (colleague satisfaction survey)	
Akhtar, 2010	Impact Assessment	Full implementation	Written/standardized exam	Rotation: 1
Ali, 2013*	pilot of tool / curriculum	Pilot	Patient survey	
An-Grogan, 2013*	Comparative study	Correlation study	Simulation-based	
Barlas, 2011*	Correlation study	Pilot	Written/standardized exam, Other (faculty assessment)	Annual: 4
Barsuk, 2009	Comparative study	Pilot	Simulation-based	
Beeson, 2006	Tool description	Full implementation	Written/standardized exam	Ever: "Multiple times"
Blouin, 2006	pilot of tool / curriculum	Pilot	Other (visual stimulation test)	Ever: 1 (in PGY5)
Bohrn, 2014*	pilot of tool / curriculum	Pilot	Direct observation (novel tool: checklist of behaviour and communication skills)	
Bounds, 2013	Impact Assessment	Pilot	Oral/verbal exam	One-off: 1
Brazil, 2012	Impact Assessment	Pilot	Direct observation (mini CEX)	Rotation: 4
Burnette, 2009	Curriculum description	Full implementation	Written/standardized exam	
Carrière, 2009	pilot of tool / curriculum	Pilot	Other (script concordance test)	
Chan, 2014*	pilot of tool / curriculum	Pilot	Direct observation (novel tool: McMaster Modular Assessment Program "McMAP")	Annual: 143 (average)
Christian, 2012*	Correlation study	Correlation study	Written/standardized exam, Simulation-based	Rotation: 2
Clark, 2010*	Curriculum description	Full implementation	Simulation-based	
Cloutier, 2013*	tool development / validation	Pilot	Simulation-based	Monthly: 2
Cooper, 2012*	Correlation study	Full implementation	360-degree/multisource feedback	Monthly: 2
Datta, 2012*	Correlation study	Correlation study	Written/standardized exam, OSCE	
Dorfsman, 2009	Curriculum description	Full implementation	Direct observation (novel tool: adapted SDOT tool)	Ever: 1 (PGY2)
Flowerdew, 2012	tool development / validation	Pilot	Direct observation (novel tool: behaviour marker system)	
Franc, 2012	Impact Assessment	Pilot	Simulation-based	One-off: 2
Frederick, 2011	Correlation study	Correlation study	Written/standardized exam, Oral/verbal exam, Other (clinical productivity)	

Gallagher, 2013*	tool development / validation	Pilot	Direct observation (novel tool: resident-resident feedback tool)	
Girzadas, 2007	tool development / validation	Pilot	Simulation-based	One-off: 0
Hauff, 2014	Tool description	Full implementation	Direct observation (novel tool: milestone-based clinical skills assessment tool), Simulation-based, Other (EM milestones global evaluation form)	Ever: 4 (PGY1)
Hogan, 2012*	Impact Assessment	Pilot	Written/standardized exam	One-off: 2
Howes, 2011*	Comparative study	comparative study	Other (oral case presentation evaluation scale)	Monthly: 4.5 average
Ilgen, 2011	Curriculum description	Full implementation	Direct observation (novel tool: assessment based on resident-tailored learning objectives), 360-degree/multisource feedback	Rotation: weekly
Jang, 2013	Comparative study	comparative study	Procedure log, Other (comparison to gold standard)	Ever: 9.4 average (PGY2-5)
Jhun, 2014*	pilot of tool / curriculum	Pilot	Simulation-based, OSAT	One-off: 1 per relevant milestone (PGY1: 2; PGY2: 6; PGY3 & PGY4: 3)
Kassam, 2014	tool development / validation	Full implementation	ITER/end of rotation assessment	Rotation: 1
Kim, 2009	tool development / validation	Pilot	Direct observation (novel tool: Ottawa critical resource management (CRM) checklist), Simulation-based, Other (global rating scale)	
Kusmiesz, 2011*	tool development / validation	Pilot	Patient survey	
Kyaw, 2012	tool development / validation	Pilot	Simulation-based, OSAT, Other (global rating scale)	
LaMantia, 2009	tool development / validation	Pilot	Direct observation (SDOT)	One-off: 1
Ledrick, 2009	Correlation study	Pilot	Written/standardized exam	Annual: 1
Ledrick, 2013*	Correlation study	Correlation study	Written/standardized exam, Other (didactic conference attendance)	
Lee, 2010*	Impact Assessment	Pilot	Written/standardized exam, Direct observation (novel tool: performance assessment tool for ultrasound)	
Lee, 2012	Correlation study	Correlation study	No tool used to assess cost knowledge (based on survey of recent graduates)	
Leech, 2013*	Impact Assessment	Pilot	OSCE	Ever: 4 (2x in PGY1 & PGY3)
Leone, 2011*	Impact Assessment	Pilot	Written/standardized exam, Direct observation (novel tool: questionnaire), Simulation-based	
Lifchez, 2012	tool development / validation	Pilot	Written/standardized exam	Rotation: 3 (start, end, 1 year after)
Mamtani, 2014*	tool development / validation	Pilot	Daily encounter cards	Rotation: daily
Marinelli, 2012*	Curriculum description	Pilot	Reflective portfolio	
McGrath, 2014*	Tool description	Pilot	Simulation-based	
McIntosh, 2012	Curriculum description	Full implementation	Oral/verbal exam, Reflective portfolio	Rotation: 2
McLaughlin, 2007	Curriculum description	Pilot	Written/standardized exam, OSCE, Direct observation (SDOT), Simulation-based, Procedure log, OSAT	
Minnigan, 2012*	pilot of tool / curriculum	Pilot	Other (novel ranking test)	

Motov, 2011	Curriculum description	Full implementation	Written/standardized exam, OSAT, Others (pre- and posttests; customized SDOT-PAIN)	Rotation: 2 weekly (total 37)
Murray, 2014*	Correlation study	Correlation study	360-degree/multisource feedback	
Nelson, 2013*	Comparative study	comparative study	Written/standardized exam, Oral/verbal exam, Simulation-based	Annual: 2
Noble, 2007	Impact Assessment	Pilot	Written/standardized exam	
Noeller, 2008	Curriculum description	Full implementation	Written/standardized exam, Simulation-based	Rotation: 2
O'Connor, 2014*	pilot of tool / curriculum	Pilot	Daily encounter cards	Rotation: Daily
Pavlic, 2014*	Curriculum description	Full implementation	360-degree/multisource feedback	
Reisdorff, 2006	pilot of tool / curriculum	Pilot	360-degree/multisource feedback, Other (Semanti differentiation scale)	
Ryan, 2010	Curriculum description	Full implementation	Direct observation (novel tool: assessment of competencies during a single patient encounter), ITER/end of rotation assessment (same tool but globally applied)	Annual: 84 (average)
Sampsel, 2014*	Curriculum description	Full implementation	Oral/verbal exam, Direct observation (novel tool: "direct observation"), Daily encounter cards, Other (targeted clinical encounters)	Rotation: 1/3 of shifts
Samuel, 2009	Impact Assessment	Pilot	Direct observation (novel tool: checklist for task completion)	One-off: 1
Scher, 2011	pilot of tool / curriculum	Pilot	Written/standardized exam	One-off: 1
Schwaab, 2011	tool development / validation	Pilot	Oral/verbal exam, Simulation-based	
Shih, 2013*	Correlation study	Full implementation	OSCE	
Sullivan, 2009	Curriculum description	Full implementation	Direct observation (novel tool: communication skills checklist), Other (videotape-facilitated self assessment)	
Thundiyil, 2010	Correlation study	Correlation study	Written/standardized exam	Annual: 1
Wagner, 2013*	Curriculum description	Full implementation	OSAT	Annual: 1
Wallenstein, 2010	Correlation study	Full implementation	OSCE, Direct observation (mini CEX), Direct observation (SDOT)	Annual: 1
Williams, 2009	tool development / validation	comparative study	Simulation-based	
Wittels, 2013*	tool development / validation	Correlation study	Simulation-based	
Zabar, 2009	pilot of tool / curriculum	Pilot	Other (unannounced standardized patients)	One-off: 2

Note:

* = abstract only

ITER = In-Training Evaluation Report

Mini-CEX = Mini-Clinical Evaluation Exercise

OSAT = Objective Structured Assessment of Technical skills

OSCE = Objective Structured Clinical Exam

SDOT = Standardized Direct Observation Tool
